# Game over? examining associations between video game play and visual and auditory spatial ability

**DOI:** 10.3389/fpsyg.2026.1752198

**Published:** 2026-04-07

**Authors:** Paul Pasescu, Daniela E. Aguilar Ramirez, Zitong Wu, Claudia L. R. Gonzalez

**Affiliations:** Department of Kinesiology and Physical Education, University of Lethbridge, Lethbridge, AB, Canada

**Keywords:** audiospatial ability, spatial cognition, video game genres, video-games, visuospatial abilities

## Abstract

Spatial ability is a strong predictor of entry into STEM fields and broader academic achievement and may relate to environmental experiences. Video games have been proposed as one such experience; however, findings remain mixed, particularly across action and non-action genres. The present study aims to investigate this relationship, specifically whether self-reported video game frequency predicts visual and auditory spatial ability. Participants (*N* = 53, *n* = 23 female) completed three spatial assessments: the Mental Rotation Test (MRT), and two ecologically valid tasks, one visual, the Brick-Building Task (BBT), and one audio spatial task, the Audio-Corsi. Gaming experience was assessed by genre, using a list of 13 categories encompassing both action and non-action games. Regression analyses revealed no significant associations between action or non-action video games and the visual or auditory tasks [βs ranged from −0.01 to 1.39; 95% CIs ranged from (−1.68, 1.98)]; all *p*s > 0.1. The results are discussed in relation to the importance of examining both, visual and auditory spatial processes using ecologically valid measures, that can contribute to a more nuanced understanding of how gaming experience may relate to spatial cognition.

## 1 Introduction

Spatial ability is defined as the capacity to mentally manipulate, rotate, and transform objects in space (Shepard and Metzler, 1971). These skills are essential in many real-world contexts, particularly in the umbrella of science, technology, engineering, and mathematics (STEM). Its importance is demonstrated by longitudinal research, such as Tian et al. (2023), who showed that spatial skills measured through a block-design test in 4th graders significantly predicted their likelihood of pursuing math-intensive STEM majors nearly a decade later, with high performers showing approximately a 50% increase in enrollment rates. Similarly, Gold et al. (2018) found that among 345 undergraduate geology students, higher spatial ability scores were positively associated with STEM motivation, standardized test performance, and the number of science courses completed. These findings illustrate the critical role that spatial ability plays in predicting and supporting long-term success and engagement in STEM fields.

Research has demonstrated that spatial abilities are not fixed; rather, they can be shaped through environmental exposure and training (Uttal et al., 2013; Yang et al., 2020). For instance, Ferrara et al. (2011) found that guided block play between young children and parents contributed to stimulation and development of spatial language and reasoning. Building on this idea, research have also identified video games as a potential tool for enhancing spatial abilities. Reviews and meta-analyses have generally supported this possibility, suggesting that video game play can positively influence aspects of spatial cognition (Spence and Feng, 2010; Uttal et al., 2013; Wang et al., 2016; Bediou et al., 2018). In particular, action video games have been proposed to enhance spatial processing because they place high demands on visual monitoring and attentional control. Supporting this idea, Feng et al. (2007) demonstrated that as little as 10 hours of action video game play (distributed across 1–2 h sessions over 4 weeks) significantly improved performance on a mental rotation task, one of the most widely used measures of spatial cognition.

However, the potential for video games to enhance spatial skills appears more nuanced. Despite evidence suggesting positive effects, a substantial body of research reports little or no reliable impact of video game play on broader cognitive abilities, including spatial skills (Unsworth et al., 2015; Sala et al., 2018). For instance, Unsworth et al. (2015), using a broad cognitive assessment battery, found no significant association between video game engagement and spatial working memory. Similarly, a meta-analysis by Sala et al. (2018) concluded that video game training produced minimal or non-significant improvements across cognitive domains, including spatial cognition. Together, these findings highlight ongoing debate regarding the robustness and generalizability of video game–related cognitive benefits (Green and Bavelier, 2007; Redick et al., 2017).

Part of this ongoing debate may reflect variability in factors such as the frequency of video game play and the types of game genres examined (Spence and Feng, 2010; Bediou et al., 2018; Choi et al., 2020). As Uttal et al. (2013) note, although some studies report no improvements in spatial skills, their findings suggest that transfer effects may emerge when training intensity or experience is sufficient. Importantly, evidence indicates that effects differ across game genres. Action video games appear to produce stronger effects on spatial cognition (see Wang et al., 2016 for a meta-analysis), whereas non-action games generally show little or no benefit (Bediou et al., 2018). For example, findings indicate that action video games may confer specific advantages, improving more basic visuospatial processes (e.g., spatial attention, spatial perception, and contrast sensitivity; Green and Bavelier, 2003, 2007; Li et al., 2009), alongside more complex spatial skills as mental rotation (Feng et al., 2007). Together, this evidence suggests that previously reported cognitive benefits may be context-dependent rather than broadly generalizable across gaming contexts.

While much of the literature has focused on visual-spatial abilities, emerging research has begun to explore whether video games (particularly action video games) might also influence auditory spatial abilities. Auditory spatial abilities refer to the capacity to perceive, represent, and remember the location and movement of sound sources (Blauert, 1997; Arnott et al., 2005). Although, both auditory and visual-spatial abilities involve the construction of internal spatial representations, they rely on different sensory inputs and cue-processing mechanisms, making it important to examine whether gaming-related effects extend across sensory modalities. Wu et al. (2021) found that action video game players showed advantage in orienting with distracting stimuli in an auditory attention network task. Similarly, action video game players in Föcker et al. (2022) performed better shifting between focused and divided auditory attention in an attention-demanding auditory spatial task. Together, these studies suggest that action video game experience may extend beyond visual processing to confer advantages in the auditory spatial domain. However, the evidence is not unequivocal. Not all findings converge on this conclusion. For example, Stewart et al. (2020) reported no association between action gaming and auditory performance when assessing attention and reaction time across multiple speech-in-noise tasks. These mixed results indicate that any potential auditory benefits of action gaming may be task-specific or dependent on the particular cognitive processes engaged.

Therefore, the conflicting evidence (spanning both visual and auditory spatial domains and varying across frequency of play and different video game genres) indicate the need for more rigorous and nuanced empirical research on the relationship between video game playing and spatial cognition. This research could have important implications for educational strategy innovations and potential optimization of games for enhancing cognitive function.

In the current study we combined a genre-based video game experience questionnaire with a battery of sensory-specific spatial assessments. The questionnaire included game play frequency across 13 distinct genres, allowing for a deeper understanding of genre-specific effects. Spatial ability was assessed using three tasks: the Mental Rotation Test (MRT; Shepard and Metzler, 1971) and brick building task (BBT; Aguilar Ramirez et al., 2020) for visual-spatial performance, as well as the Audio-Corsi task (Setti et al., 2022; Aguilar Ramirez et al., 2025) for auditory spatial ability. Rather than relying solely on traditionally used computer- or paper-based tests, this study incorporates hands-on sensorimotor spatial tasks such as the BBT and Audio-Corsi to better reflect real-world spatial demands. By doing this, we aim to strengthen the ecological validity of video gaming as a practical tool for spatial ability development. Given the mixed results in the existing literature linking video game play with enhanced visual and auditory spatial abilities, the present study adopts a non-directional hypothesis. In other words, rather than predicting a positive or negative association between gaming experience and spatial performance, we simply test for the presence of any systematic relationship acknowledging that prior findings do not justify a clear directional expectation.

## 2 Methods

### 2.1 Participants

Recruitment occurred in three phases across a full academic year. In the initial phase, participants were recruited from a 2nd-year undergraduate neuroscience course, where individuals who self-identified as regular video game players (“gamers”) were specifically invited to participate. From this, 23 participants were recruited (*n* = 11 male, *n* = 12 female). In the second phase, another undergraduate neuroscience class was approached, this time requesting only those who self-identified as non-gamers. Outcomes of this recruitment were 2 participants (*n* = 2 male). Due to lack of engagement, the final phase of recruitment opened participation to all students through word-of-mouth around campus and through the University of Lethbridge Department of Psychology's participant management system (Sona Systems), which allowed students to received course credit for their participation.

Across all phases, 53 undergraduate students aged 18–35 were recruited (23 females, 30 males). Participants from all recruitment phases were combined into a single sample, and gaming experience was treated as a continuous individual-differences variable rather than as a categorical grouping factor. Although this phased recruitment strategy allowed for variation in gaming experience, it may limit representativeness and introduce potential selection bias, which should be considered when interpreting the findings. Ethics approval was granted by the University of Alberta.

Because the hypothesis was non-directional, a two-tailed sensitivity analysis was conducted in G^*^Power 3.1 (Faul et al., 2009). With *N* = 53, α = 0.05, and 80% power, the study was sensitive to correlations of approximately *r* ≥ 0.37, indicating adequate power to detect moderate-to-large effects but limited sensitivity to small effects.

### 2.2 Spatial Assessment

#### 2.2.1 Mental rotation test

**Figure 1:**
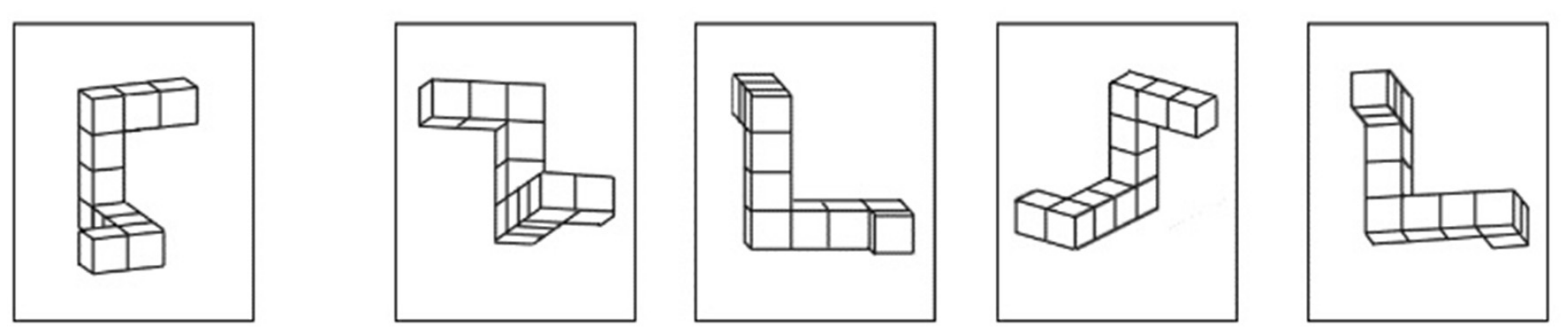
The mental rotation test. An example trial from the Mental Rotation Test (MRT) is presented. The figure on the left depicts the target and is compared to the 4 figures on the right. The participant's task is to identify which 2 of the 3 figures are rotated versions of the target. In this case, the 2nd and 3rd figures are the correct match.

The MRT task is described as a “pure” visuo-spatial assessment of mental rotation ability (Shepard and Metzler, 1971). A total of 24 trials were used in testing (see Figure 1), split up into two sets of 12 questions. Participants were required to select the two figures that matched the target (Peters et al., 1995).

#### 2.2.2 Brick-building task

**Figure 2:**
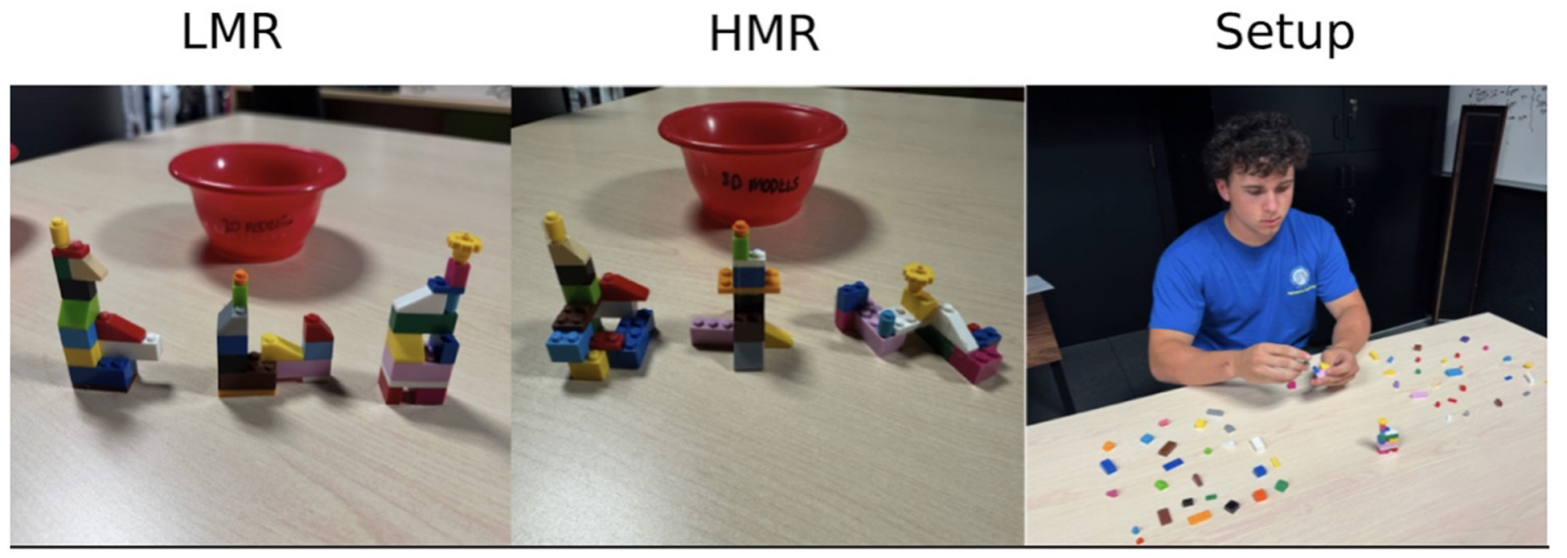
BBT set up. LMR and HMR model sets, as well as testing setup with distractor bricks.

The BBT was used to assess visual search and mental rotation abilities, it included a Low Mental Rotation (LMR) and High Mental Rotation (HMR) condition comprised of three trials (Aguilar Ramirez et al., 2020; see Figure 2). In the LMR condition, bricks were configured to be visible from a single viewpoint, thereby reducing the need for mental rotation. By contrast, the HMR condition used identical types and quantities of bricks but arranged them to emphasize three-dimensional depth, requiring participants to inspect and interpret the structures from multiple perspectives and thus increasing mental rotation demands (see Figure 2 and Aguilar Ramirez et al., 2020, for a detailed description of the task). He LMR and HMR conditions were designed to systematically manipulate mental rotation demands while controlling for materials, with prior work demonstrating expected performance differences between conditions (Aguilar Ramirez et al., 2020, 2021, 2022). The BBT has been previously used as a hands-on measure of spatial ability, with performance showing moderate-to-large correlations with the MRT (Aguilar Ramirez et al., 2020, 2021, 2022).

At the beginning of the BBT task, 60 unique bricks were arranged in a pseudo-random manner in front of the participant (30 on the left and 30 on the right). Among these, 36 bricks were specific to the models (12 pieces for each of the three target models, evenly split between both sides), while the remaining 24 served as distractors (12 on each side). After each trial, the number of available bricks decreased by 12, reflecting the pieces used to reconstruct the model. The sequence of conditions and trials (i.e., models) was randomized for every participant.

#### 2.2.3 Audio-corsi task

**Figure 3:**
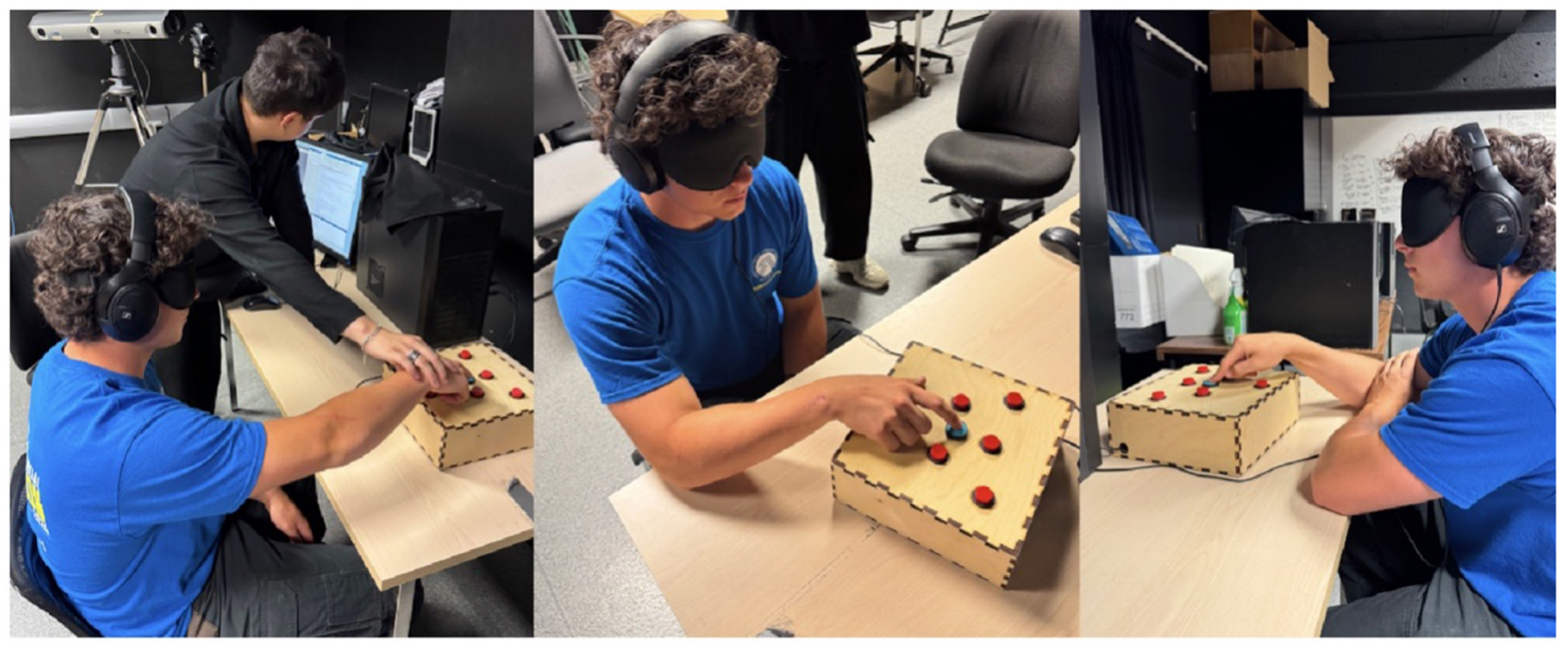
The Audio- Corsi task set up. Participants are seated blindfolded, guided by a test instructor around the testing box that is recorded via the MATLAB program.

The Audio-Corsi task was adapted from Setti et al. (2022; see Figure 3) and was used to assess auditory spatial working memory. Participants were blindfolded and wore headphones while interacting with an Arduino-based wooden keyboard featuring six red buttons and one central blue reference button (Figure 3). The keyboard was connected to a computer via USB and operated using MATLAB software. Each red button corresponded to one of six spatialized sound locations. During the task, participants heard sequences of identical pink noise stimuli, spatialized binaurally using the 3D Tune-In Toolkit Binaural Test App (Cuevas-Rodríguez et al., 2019; Figure 3). Participants completed both Forward and Backward conditions, in which they were asked to recall the sequence of sounds in the same order or in reverse, respectively. The task consisted of a maximum of 36 sequences per condition, progressing in length from 2 to 9 sounds. Each sequence length included four trials, and participants could only advance to the next sequence length after correctly completing at least one trial at the current level. For more detailed information on the hardware, software, and task development, see Setti et al. (2022).

### 2.3 Procedure

Participants were first asked to read and sign a consent form and a personal information form. The personal information form captured self-reported: sex, gender, dominant hand, and undergraduate degree program. Task order (MRT, BBT, or Audio-Corsi) was counterbalanced between participants and the order of the trials within each task (e.g., which model of the BBT was presented first) was randomized for each participant. For the MRT, participants were given an overview before testing and given a practice question to prepare with. Once familiar, participants were given 3 min to complete as many trials as possible within a set of 12 MRT trials, followed by a 3-min break, and another 3 min to complete a second set of 12 questions.

In the BBT, participants were seated at a table positioned directly in front of the center of the brick display (Figure 2). Participants were presented with a pre-built model and instructed to replicate it with speed and accuracy, using the bricks scatter on the tabletop before them. Once presented with the model, they were free to move and rotate the model to aid in replication. Upon replication, participants were required to give verbal confirmation to denote completion.

For the Audio-Corsi task, participants were blindfolded, fitted with headphones, and physically guided around the parameters of the testing apparatus (see Figure 3). Participants were then given 3 min to familiarize themselves with all buttons and their respective sounds, followed by a sound check, with any clarification of sounds made and errors corrected. The forward condition was explained, supplemented by a verbal example, and then performed. This was followed by another sound check and the same procedure for completion of the backwards condition.

Upon completing all three tasks, participants were given two experience-related questionnaires: one assessing their self-reported comfort playing with toy bricks (e.g., LEGO), and another evaluating their comfort and frequency of video game play with different genres. The toy-brick experience questionnaire asked participants to self-report their comfort with toy-bricks (i.e., “how comfortable are you at manipulating toy-bricks [LEGO or similar]” on a scale of 1–10 [1 being not comfortable and 10 being completely comfortable])”; Aguilar Ramirez et al., 2020). A modified version of this questionnaire was used for the gaming experience questionnaire, asking participants to self-report comfort, and frequency of engagement in 13 video game genres. Genres of video games were adapted from Starosta et al. (2024), who conducted a systematic review of game genre classifications across psychological research. For the questionnaire, the following 13 genres were adapted: 1st/3rd-Person Shooter (e.g. Call of Duty), Sports/Driving (e.g. FIFA), Real-Time Strategy (RTS; e.g. Starcraft II), Multi-Player Online Battle Arena (MOBA; e.g. Dota), Turn-Based Strategy (e.g. Civilization), Role-Playing (e.g. Fallout II), Action-Role-Playing (e.g. Mass Effect 3), Adventure (e.g.. Life is Strange), Action-Adventure (e.g. Assassin's Creed), Mini-games (e.g. Angry Birds), Puzzle (e.g. Portal 2), Music (e.g. Guitar Hero), and Sandbox (e.g. Minecraft). A 10-point Likert scale was used to report engagement with each genre, ranging from 0 (never) to 10 (daily play). Refer to appendix A to see the full questionnaires.

These questionnaires were designed to measure pre-existing experience variables relevant to spatial performance rather than task-induced responses. Items asked participants to report their typical or historical experience levels. Questionnaires were administered after task completion to minimize potential priming effects that could influence spatial task strategy or performance. Responses were therefore interpreted as individual-difference predictors rather than outcomes of task engagement.

### 2.4 Data analysis

For the MRT task, participants were evaluated based on the sum of the number of correctly answered questions of the total 24 questions. A correct score was assigned to questions only when both correct answers were selected, thus a higher score reflected better performance.

To evaluate the BBT, model replication time was recorded, and the number of grabbing errors, building errors, and missing errors were counted and summed for a total error score for each LMR and HMR model. These measures were analyzed using video recordings of each trial.

For evaluation of the Audio-Corsi, the maximum stage achieved (span) and number of correct sequences achieved were recorded. Two composite scores were used for evaluation, calculated as the products of span and number of correct sequences for both the forward and backward conditions, thus a higher score reflected better performance.

To examine whether different genres of gaming experience were differentially associated with spatial performance, genres were grouped into Action and Non-Action categories based on prior cognitive-demand frameworks suggesting that action games typically impose high perceptual load, rapid visuomotor coordination, and sustained attentional monitoring (Spence and Feng, 2010; Bediou et al., 2018; Wang et al., 2016). This distinction was therefore grounded in hypothesized cognitive demands rather than taxonomic similarity alone.

ChatGPT (GPT-4.0) was used as a practical tool to assist in applying these predefined criteria consistently across the 13 genres. The Action category included FPS, Sports/Driving, RTS, MOBA, Action-Role-Playing, Adventure, and Action-Adventure games. The Non-Action category included Turn-Based Strategy, Role-Playing, Mini-games, Puzzle, Music, and Sandbox games. Because genre categories are inherently heterogeneous, the action/non-action grouping was intended to approximate differences in attentional and spatial processing demands rather than to provide a strict taxonomic classification. Alternative groupings were explored, and these did not materially alter the overall pattern of results.

Pearson correlation coefficients were calculated between the spatial ability tasks (MRT, LMR and HMR conditions of the BBT, and forward/backward total scores of the Audio-Corsi) and questions in the self-reported questionnaires: (1) comfort with toy-brick play, (2) comfort with video game play, (3) and frequency of video game play on Action and Non-Action categories. To control for inflation of Type I error due to multiple testing, p-values were adjusted using a Bonferroni correction (α adjusted = 0.05/ *N* comparisons). In this case significant correlations were those smaller than *p* = 0.0045 (0.05/11).

To investigate whether any of the self-reported questions of the questionnaires predicted performance on the spatial ability tasks, separate multiple regression analyses were conducted for each spatial ability task. Each task was treated as a distinct dependent variable, with the same set of predictors (self-reported answers) included in all models (entered simultaneously). The alpha level for the multiple regression analyses were 0.05. IBM SPSS Statistics (Version 29) was used for all analyses.

## 3 Results

### 3.1 Descriptive statistics

Descriptive statistics for all study variables are presented in Table 1. Means, standard deviations, and ranges are reported for spatial ability measures, gaming categories, and comfort questionnaires.

**Table 1:** Descriptive statistics for all variables included in the analyses.

Dependent variable	M	SD	Min-max	Skewness
LMR (time)	54.02 s	17.48 s	31.67–103	0.99
LMR (errors)	2.08	2.81	0–15	2.65
HMR (time)	87.46	35.49	41.67–205	1.51
HMR (errors)	2.68	2.06	0–9	0.96
Audio-Corsi forward (score)	4.06	1.45	0.56–6.65	−0.25
Audio-Corsi backward (score)	3.95	1.37	0–6.98	−0.27
MRT (score)	8.98	4.68	0–21	0.39
Lego comfort	6.51	3.16	1–10	−0.65
Gaming comfort	7.28	3.09	1–10	−0.90

### 3.2 Correlation analysis

**Figure 4:**
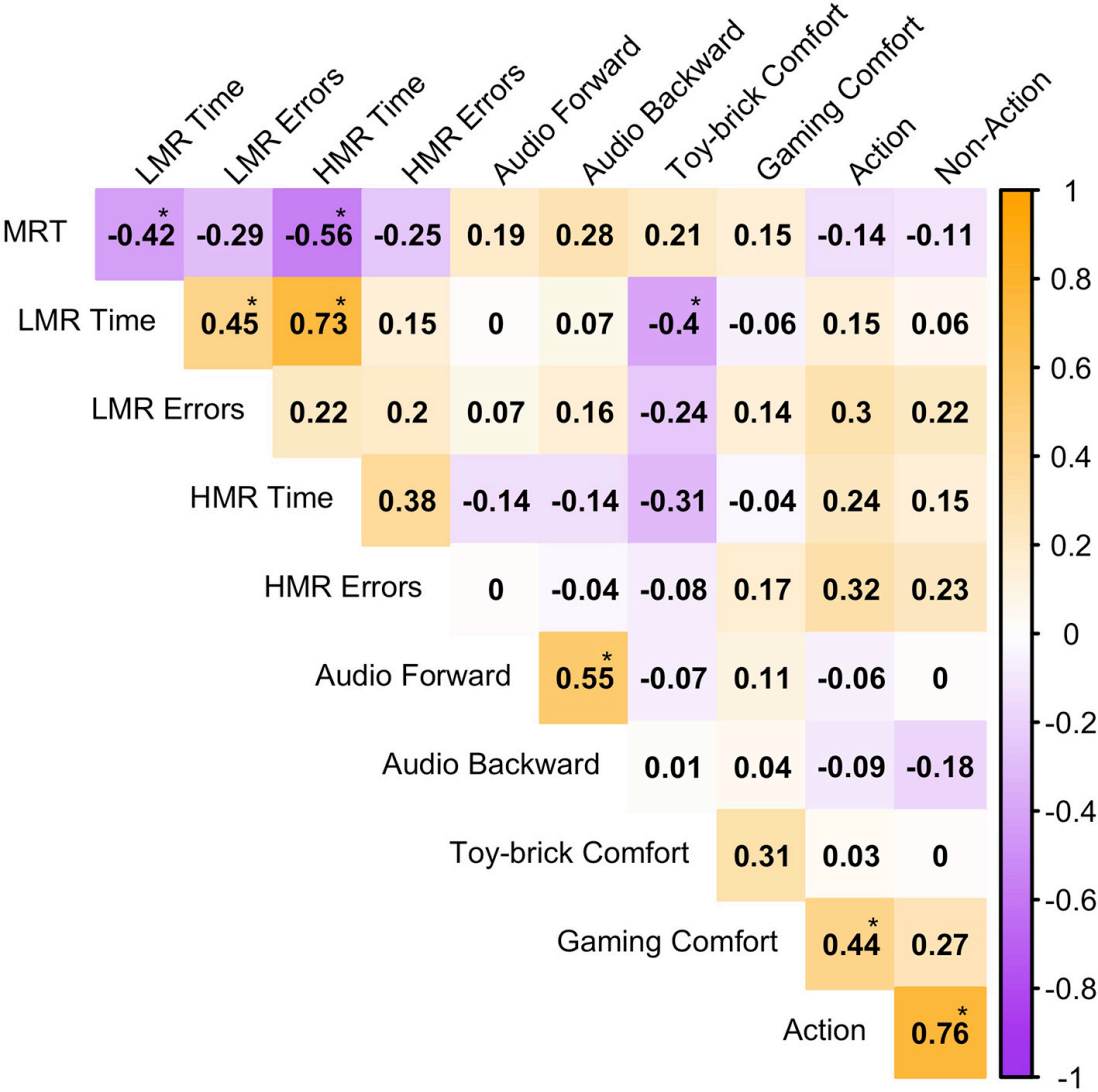
Correlation matrix for the dependent variables (spatial assessments) and two-experience related questionnaires (toy-brick and video game comfort). Significance was evaluated at *p* <0.0045^*^ following Bonferroni correction for multiple comparisons.

Given prior evidence that action video games influence cognitive function, we grouped video game genres (adapted from Starosta et al., 2024 and classified using AI) into two categories: Action (FPS, Sports/Driving, RTS, MOBA, Action-Role-Playing, Adventure, Action-Adventure) and Non-Action (Turn-Based Strategy, Role-Playing, Mini-games, Puzzle, Music, Sandbox). Figure 4 shows the correlations with these two broader categories. After applying corrections for multiple comparisons, only the association between Gaming comfort was correlated (positively) with Action game play (*r* = 0.44, *p* < 0.001). No other significant relationships were identified for either gaming category.

We further conducted multiple regression analyses using the Action and Non-Action categories as predictors to assess their unique effects on spatial task performance while controlling for overlap among genres.

### 3.3 Regression analyses

Separate regression analyses were conducted for each spatial ability task (LMR and HMR from the BBT, forward and backward total scores from the Audio-Corsi, and MRT), each as a distinct dependent variable. The same predictors (Action, non-Action, toy-brick comfort, and Gaming comfort) were included in all models. The only significant model [F _(4.49)_ = 2.7, *p* < 0.05, *R*^2^ = 0.18) was for LMR time (see Table 2). Toy-brick comfort was the only predictor of LMR time performance (*p* < 0.01). Thus, the higher toy-brick comfort the faster completion times in the LMR task. Note, that none of the other models yielded significant results (see Table 2).

**Table 2:** Results of the regression analyses.

Predictor	LMR (*t*) β [95% CI]	LMR (*e*) β [95% CI]	HMR (*t*) β [95% CI]	HMR (*e*) β [95% CI]	ACF (*s*) β [95% CI]	ACB (*s*) β [95% CI]	MRT (*s*) β [95% CI]
Action	0.29[−0.19, 0.87]	0.27[−0.70, 4.0]	0.36[−0.21, 1.98]	1.39[−0.02, 0.11]	−0.26[−0.07, 0.02]	0.34[−0.04, 0.05]	−0.25[−0.23, 0.07]
Non-action	−0.17[0.92, 0.38]	−0.01[−0.04, 0.14]	−0.11[−1.68, 0.99]	−0.02[−0.08, 0.75]	0.12[−0.04, 0.7]	−0.28[−0.09, 0.02]	0.03[−0.17–0.20]
Toy-brick comfort	−0.06[−3.7, −0.58]^*^	0.12[−0.50, 0.02]	−0.29[−6.47,−0.13]	−0.09[−0.25, 0.13]	−0.14[−0.21, 0.8]	−0.03[−0.15, 0.12]	0.19[−0.16–0.71]
Gaming comfort	−0.38[−2.10, 1.44]	−0.27[−0.18, 0.40]	−0.09[−4.69, 2.61]	0.08[−0.17, 0.27]	0.22[−0.06–0.26]	0.05[−0.13–0.17]	0.19[−2.1, 0.79]

*t*, time; *e*, errors; *s*, score; ACF, Audio-Corsi Forward; ACB, Audio-Corsi Backward; β, Standardized coefficients beta. CI, Confidence Intervals ^*^Only significant model.

## 4 Discussion

Utilizing a gaming questionnaire and a combination of visual and auditory spatial assessments, the present study aimed to investigate the association between self-reported video game activity and spatial ability outcomes. In the current sample no association between Action or Non-Action video game play and spatial ability, either visual or auditory, was found.

Although our results may diverge from some previous literature, it is important to note that prior studies typically relied on correlation analyses or analyses of variance to compare groups of “gamers” vs. “non-gamers” (e.g., Unsworth et al., 2015; West et al., 2020; Bailey et al., 2010; Feng et al., 2007). Such a dichotomous classification was not feasible in the present study. Despite a specific call for non-gamers, the vast majority of participants self-reported meaningful engagement with video game playing. Moreover, by including 13 different genres, we likely reduced the possibility of identifying individuals with no gaming exposure whatsoever. More broadly, this might reflect a structural challenge in contemporary university samples in developed countries, where true “non-gamers” may be increasingly rare or virtually absent.

The present data did not reveal reliable evidence that self-reported video game history predicted spatial performance. The only significant predictor was self-reported toy-brick comfort, which was associated with faster completion times in the LMR (*p* < 0.01) condition. These findings replicate our previous work with young adults (Aguilar Ramirez et al., 2020). We also replicated the previous result of an association between the MRT and the BBT (see Figure 4 of the current study, and Aguilar Ramirez et al., 2020, 2021, 2022); in the current study higher MRT scores were significantly correlated with shorter completion times on both the LMR (*r* = −0.42, *p* < 0.004) and HMR (*r* = −0.56, *p* < 0.001) conditions. These converging results suggest that the MRT and BBT draw on shared spatial mechanisms, linking mental rotation ability to performance on a task requiring complex visuospatial construction. Taken together, the findings suggest that self-reported video game experience was not reliably associated with visual or auditory spatial performance in the present sample. Instead, individual differences were most evident when prior experience overlapped with the demands of the specific task, as in the case of toy-brick familiarity and the BBT.

Importantly, these findings should be interpreted within the broader methodological landscape of the literature. Although the present study aligns with correlational work reporting limited associations between gaming and spatial outcomes, the field also includes randomized training studies demonstrating causal effects of video game play on spatial cognition (e.g., Feng et al., 2007). Because the current study employed a correlational design, it cannot provide causal evidence for or against gaming-related cognitive effects. Instead, our results are best viewed as complementary to experimental research, suggesting that naturally occurring variation in self-reported gaming exposure within contemporary university samples may not strongly predict individual differences in spatial performance.

One strength of the present study is the inclusion of the Video Game Experience Questionnaire with thirteen genres. Prior research has often collapsed gaming exposure into broad categories (e.g., gamer vs. non-gamer) or focused narrowly on subsections of action games, particularly shooter titles (Feng et al., 2007). In contrast, our design incorporated a detailed breakdown of thirteen widely recognized genres. These categories were adapted from compilations of video game genres used in psychological research (Starosta et al., 2024) and paired with examples of well-known titles to enhance clarity and accuracy for participants. By capturing experience across both action and non-action classifications, the questionnaire allowed for a more nuanced examination of potential sub-genre associations with spatial abilities. Despite this robust classification, few and “small” correlation were found between genre and spatial outcomes. This suggests that the spatial effects of gaming may not be strongly genre-dependent, or alternatively, that self-reported questions do not adequately capture the complexity and intensity of gameplay required to influence spatial abilities.

Another strength of this study is its examination of visual spatial cognitive demands central to action video game play, alongside the less-explored auditory spatial domain. Action games are known to engage visuospatial processes such as opponent tracking, peripheral attention, and mental rotation, and they also place demands on auditory skills through sound localization and cue processing (Bediou et al., 2018; West et al., 2020). To our knowledge, this is the first study to assess associations between gaming and auditory spatial working memory. The absence of measurable effects in the present study suggests that potential transfer effects may not have been detectable using self-reported gaming measures within this sample. Importantly, this pattern is not unique to self-report approaches, as evidence from naturalistic visual search tasks also shows no differences in multisensory (visual–auditory) integration efficiency between action video game players and non-players (Hamzeloo et al., 2024). Together, these findings underscore the importance of continuing to investigate both visual and auditory spatial processes, particularly in naturalistic settings, as this may provide a more comprehensive understanding of how gaming experiences relate to spatial cognition.

These study carries certain limitations important to acknowledge. First, is the use of a self-reported questionnaire as the only measure of participant video game experience. No prescription of video gameplay was applied to the study (unlike Feng et al., 2007), restricting our evaluation to subjective self-reported measures of gaming experience, which has potential to be misaligned with objective gaming experience history. Second, the structure of the questionnaire, imposed constraints on the precision of measurement. Comfort with gaming and frequency of play were assessed on 10-point Likert scales, which provide only coarse estimates of experience. For example, genre frequency was measured on a scale from 1 (Never) to 10 (Daily), which captures how often participants play but not how long they play in each session or their cumulative history with a genre. As a result, heavy and casual players who both report “daily play” cannot be distinguished. These two limitations may have obscured meaningful variation in gaming experience and restricted our ability to detect fine-grained associations between specific genres and spatial abilities. Furthermore, by assessing only genre-specific frequency of engagement, the questionnaire did not capture additional dimensions of gaming expertise such as session duration, lifetime years of experience, skill level, or enjoyment. Future research would benefit from incorporating multidimensional assessments of gaming exposure to better characterize expertise and its potential cognitive associations. Third, initial recruitment targeted undergraduates with specific gaming backgrounds (self-identified gamers and non-gamers) before recruitment was broadened to a general undergraduate population to achieve the planned sample size and increase variability in gaming experience. Although gaming experience was treated as an individual-differences variable in the analyses, this recruitment approach may introduce selection bias and limit the representativeness of the sample relative to the broader population. Consequently, the generalizability of the findings should be interpreted with caution. Fourth, although, the study was adequately powered to detect moderate-to-large effects, it had limited sensitivity to small associations.

A final limitation of this study is the possibility of genre misclassification when distinguishing between action and non-action games. To categorize genres reported in the experience questionnaire into action/non-action, we used ChatGPT-4.0. While these classifications were based on widely used definitions, certain titles inevitably blur category boundaries. Although we did not test every possible permutation of game groupings, we explored several alternative classifications. Importantly, none of these variations altered the overall results, suggesting that our null findings are not simply an artifact of how genres were grouped. Future research would benefit from refining measurement tools and expanding the demographics of participant sampling. In particular, the underrepresentation of females in the current sample limits the strength of conclusions regarding potential sex-specific effects. Recruiting more balanced samples, alongside specialized groups such as competitive eSports players and true non-gamers, would allow for a clearer assessment of dose–response effects and possible sex differences. Given that most university-aged individuals have some degree of gaming exposure, diversifying the sample to include a broader adult age range may increase variability in cumulative gaming experience and exposure histories, allowing a representative examination of how gaming relates to spatial abilities across adulthood. Together, these approaches would provide a more nuanced understanding of when, how, and for whom gaming experience influences spatial abilities, and whether particular spatial domains are more sensitive to gameplay than others.

## Data Availability

The raw data supporting the conclusions of this article will be made available by the authors, without undue reservation.

## Appendix A

### Gaming experience questionnaire

Please indicate how comfortable are you at playing video games on a scale form 1–10 (10 being completely comfortable, 1 being not comfortable at all).

1  2  3  4  5  6  7  8  9  10

Indicate how many times do you play 1st/3rd-person shooter games (ex. call of duty) in a week on a scale of 1–10 (1 being never and 10 being daily play).

1  2  3  4  5  6  7  8  9  10

Indicate how many times do you play Sports/Driving games (ex. FIFA) in a week on a scale of 1–10 (1 being never and 10 being daily play).

1  2  3  4  5  6  7  8  9  10

Indicate how many times do you play Real-Time Strategy games (ex. Starcraft II) in a week on a scale of 1–10 (1 being never and 10 being daily play).

1  2  3  4  5  6  7  8  9  10

Indicate how many times do you play Multi-Player Online Battle Arena (MOBA) games (ex. League of Legends) in a week on a scale of 1–10 (1 being never and 10 being daily play).

1  2  3  4  5  6  7  8  9  10

Indicate how many times do you play turn-based strategy games (ex. Civilization) in a week on a scale of 1–10 (1 being never and 10 being daily play).

1  2  3  4  5  6  7  8  9  10

Indicate how many times do you play role-playing games (ex. Fallout II) in a week on a scale of 1–10 (1 being never and 10 being daily play).

1  2  3  4  5  6  7  8  9  10

Indicate how many times do you play Action-Role-Playing games (ex. Mass Effect 3) in a week on a scale of 1–10 (1 being never and 10 being daily play).

1  2  3  4  5  6  7  8  9  10

Indicate how many times do you play adventure games (ex. life is strange) in a week on a scale of 1–10 (1 being never and 10 being daily play).

1  2  3  4  5  6  7  8  9  10

Indicate how many times do you play action-adventure games (ex. assassin's creed) in a week on a scale of 1–10 (1 being never and 10 being daily play).

1  2  3  4  5  6  7  8  9  10

Indicate how many times do you play mini-games games (ex. angry birds) in a week on a scale of 1–10 (1 being never and 10 being daily play).

1  2  3  4  5  6  7  8  9  10

Indicate how many times do you play puzzle games (ex. portal 2) in a week on a scale of 1–10 (1 being never and 10 being daily play).

1  2  3  4  5  6  7  8  9  10

Indicate how many times do you play music games (ex. guitar hero) in a week on a scale of 1–10 (1 being never and 10 being daily play).

1  2  3  4  5  6  7  8  9  10

Indicate how many times do you play sandbox games (ex. Minecraft) in a week on a scale of 1–10 (1 being never and 10 being daily play).

1  2  3  4  5  6  7  8  9  10

If unsure about a certain game genre, indicate how many times do you play other games (misc.) in a week on a scale of 1–10 (1 being never and 10 being daily play), and please specify what game below.

1  2  3  4  5  6  7  8  9  10

Game(s): _________________________________________
