# Supplementary material for: Game over? examining associations between video game play and visual and auditory spatial ability
Source: Front Psychol. 2026 Apr 7;17:1752198. doi: 10.3389/fpsyg.2026.1752198 (PMC13096050; doi:10.3389/fpsyg.2026.1752198)
Supplement: Supplementary file 1 [file Supplementary_file_1.docx]

**Appendix A**

**Gaming Experience Questionnaire**

Please indicate how comfortable are you at playing video games on a scale form 1-10 (10 being completely comfortable, 1 being not comfortable at all)

1 2 3 4 5 6 7 8 9 10

Indicate how many times do you play First/Third-Person Shooter games (ex. Call of Duty) in a week on a scale of 1-10 (1 being never and 10 being daily play).

1 2 3 4 5 6 7 8 9 10

Indicate how many times do you play Sports/Driving games (ex. FIFA) in a week on a scale of 1-10 (1 being never and 10 being daily play).

1 2 3 4 5 6 7 8 9 10

Indicate how many times do you play Real-Time Strategy games (ex. Starcraft II) in a week on a scale of 1-10 (1 being never and 10 being daily play).

1 2 3 4 5 6 7 8 9 10

Indicate how many times do you play Multi-Player Online Battle Arena (MOBA) games (ex. League of Legends) in a week on a scale of 1-10 (1 being never and 10 being daily play).

1 2 3 4 5 6 7 8 9 10

Indicate how many times do you play Turn-Based Strategy games (ex. Civilization) in a week on a scale of 1-10 (1 being never and 10 being daily play).

1 2 3 4 5 6 7 8 9 10

Indicate how many times do you play Role-Playing games (ex. Fallout II) in a week on a scale of 1-10 (1 being never and 10 being daily play).

1 2 3 4 5 6 7 8 9 10

Indicate how many times do you play Action-Role-Playing games (ex. Mass Effect 3) in a week on a scale of 1-10 (1 being never and 10 being daily play).

1 2 3 4 5 6 7 8 9 10

Indicate how many times do you play Adventure games (ex. Life is Strange) in a week on a scale of 1-10 (1 being never and 10 being daily play).

1 2 3 4 5 6 7 8 9 10

Indicate how many times do you play Action-Adventure games (ex. Assassin’s Creed) in a week on a scale of 1-10 (1 being never and 10 being daily play).

1 2 3 4 5 6 7 8 9 10

Indicate how many times do you play Mini-games games (ex. Angry Birds) in a week on a scale of 1-10 (1 being never and 10 being daily play).

1 2 3 4 5 6 7 8 9 10

Indicate how many times do you play Puzzle games (ex. Portal 2) in a week on a scale of 1-10 (1 being never and 10 being daily play).

1 2 3 4 5 6 7 8 9 10

Indicate how many times do you play Music games (ex. Guitar Hero) in a week on a scale of 1-10 (1 being never and 10 being daily play).

1 2 3 4 5 6 7 8 9 10

Indicate how many times do you play Sandbox games (ex. Minecraft) in a week on a scale of 1-10 (1 being never and 10 being daily play).

1 2 3 4 5 6 7 8 9 10

If unsure about a certain game genre, indicate how many times do you play other games (misc.) in a week on a scale of 1-10 (1 being never and 10 being daily play), and **PLEASE SPECIFY** what game below.

1 2 3 4 5 6 7 8 9 10

Game(s): _________________________________________
